# Importance of Tuberculosis Screening of Resident Visa Applicants in Low TB Incidence Settings: Experience from Oman

**DOI:** 10.1007/s44197-022-00040-w

**Published:** 2022-04-25

**Authors:** Jeffrey Singh, Seif Al-Abri, Eskild Petersen, Fatma Al Yaqoubi, Khoula Al Rahbi, Lamya Al Balushi, Fatma Al Fahdi, Asma Al Balushi, Farah Mahmmoud M. Jawad, Padmamohan J. Kurup

**Affiliations:** 1 Department of Disease Surveillance and Control, Directorate General of Health Services, Muscat Governorate, Muscat, Oman; 2 Directorate General for Disease Surveillance and Control, Ministry of Health, Sultanate of Oman, 393, 100 Muscat, Oman; 3 Institute for Clinical Medicine, Faculty of Health Science, University of Aarhus, Denmark and ESCMID Emerging Infections Task Force, Basel, Switzerland

**Keywords:** Tuberculosis, Screening, Chest X-ray, Expatriates, Low TB incidence

## Abstract

**Introduction:**

For Oman, a country targeting tuberculosis (TB) elimination, TB among expatriates is a major challenge. Thus, screening for active TB using chest X-ray was made mandatory for expatriates’ residency renewals.

**Objective:**

To estimate the incidence of bacteriologically confirmed TB and assess impact of chest X-ray based TB screening among expatriates in Muscat Governorate.

**Methods:**

Applicants for residency and renewals were mandated for chest X-ray-based TB screening in 2018. We collected data of screened subjects with radiological suspicion of TB who were subjected to further bacteriological evaluation.

**Results:**

Of 501,290 applicants screened during the study period, 436 (0.09%) had X-ray findings suggestive of TB. Among the 436, TB was confirmed in 53 (12.2%; 95% CI 9.2–15.6), giving an overall prevalence of 10.6 (95% CI 8–13.9) per 100,000 applicants (number needed to be screened 9458). Among renewals, the point prevalence of TB was 10.5 per 100,000 expatriates screened (95% CI 6.9–14.04 per 100,000), with a mean follow-up period of 11.8 years.

**Conclusion:**

Our findings are consistent with the recommendation for utilization of chest X-ray as a preferred tool for active case finding in the setting of expatriate screening. Our findings are also suggestive of the need for latent TB screening and ruling out TB prior to latent TB treatment.

## Introduction

Tuberculosis (TB) remains the world’s deadliest infectious disease and claims more than a million lives each year. Globally, the annual number of people reported to have accessed TB treatment has grown from about 6 million in 2015, to 7.1 million in 2019. National TB rates vary widely from less than five to more than 500 cases per 100,000 population per year [1]. The World Health Organization (WHO) End TB Strategy aims to end the global TB epidemic by 2035 targeting a reduction in TB incidence to below ten cases per 100,000 population per year [2]. In low-burden countries that have already reached this threshold, an action framework has been proposed by the WHO to progress rapidly toward pre-elimination (one case per 100,000 population per year) and elimination (one case per million population per year)with the WHO calling for elimination in low-burden countries by 2050 [2].

The Sultanate of Oman, a member of the Gulf Cooperation Council (GCC) is a low TB incidence country, with an annual incidence rate of eight cases per 100,000 population in 2020 and is well placed to target TB elimination [2, 3]. In 1981, the annual incidence of TB in Oman was over 90 per 100,000 population. Following rapid economic development in the 1980s and after, the incidence declined to 20 in 1991 and 10 by 2010 [3–5]. Oman has a population of 4.6 million, of which 41% are expatriates having employment visa with residence permit and not having citizenship [6]. The majority of expatriates in Oman constitute less educated, semiskilled and unskilled workers in comparison with migrants to OECD countries and a major proportion are from the Indian subcontinent. A lesser proportion of expatriates are from Middle East and North African countries as well as from Southeast Asian countries [6, 7]. These diversities could in the context of communicable diseases “lead to internationally distinct set of outcomes for the host countries, migrant workers and source countries” [7]. Active TB cases were slowly declining among Omani nationals while there is a steady increase among expatriates [3–5].This suggests that TB transmission among and from the expatriate population is problematic requiring multiple interventions as reported from other parts of world [8]. Incidence rates based on notified cases may not fully reflect the burden due to underreporting [3]. Although underreporting is expected to be low with universal access to health care in Oman and provision of free TB treatment for all including expatriates, there is high probability of non-detection and underreporting of TB among expatriates [9].

Oman is a signatory of the Moscow Declaration to End TB [10]. The TB elimination framework program for Oman has promoted public–private partnerships, community support in TB treatment, and evaluation of screening methods [3–5]. In 2017, Oman made policy changes in TB screening of expatriates through a ministerial order issued by the Minister of Health. This facilitated implementation of change in TB screening of expatriates for renewal of residence permits by replacing symptom-based screening with radiological screening. With radiological suspicion of TB, applicants were subjected for sputum microscopy, culture, and GeneXpert testing [11].

Systematic screening for active TB is a priority action area for low-burden countries [12–14].The change from symptom based to radiological screening implemented in 2018 necessitated operational studies to assess yield and also it supports local capacity building and insights to TB epidemiology in high-burden countries for future planning [3, 12, 15].

In this study, we aimed to estimate the incidence of bacteriologically confirmed TB and evaluate the performance and results of the systematic chest X-ray-based TB screening among expatriates applying for a new visa or residency renewal from 2018 to 2020 in Muscat Governorate.

## Methods

### Medical Screening Process for Expatriates

All expatriates applying for employment/residency in Oman (as in all GCC countries) are required to have a medical examination either in their country of origin (pre-arrival screening) if Gulf Approved Medical Centers Association (GAMCA) centers are present in their country or upon arrival in Oman. This should be undertaken either 3 months before arrival or within 4 weeks of entry to Oman. An expatriate who is already a resident is mandated to undergo screening for renewal of residency every 2 years. Persons below 18 years are not subjected to medical examination for issuance of residence permits.

The medical examination includes screening for infectious diseases and TB screening by history, physical examination and a chest X-ray. Those who have been fitness certified in their country of origin will be verified by physical examination alone at-entry screening. Once TB and HIV are ruled out, the visa will be granted. For renewal of residency TB screening was symptom based and X-ray screening was not mandatory prior to 2018. An electronic central database is operational and has been used for issuance of fitness certificates since 2018. Applicants detected to have an infectious disease on screening are further evaluated by a standard process which is accessible at all centers in the country.

### Intervention and Target Population

**Fig. 1:**
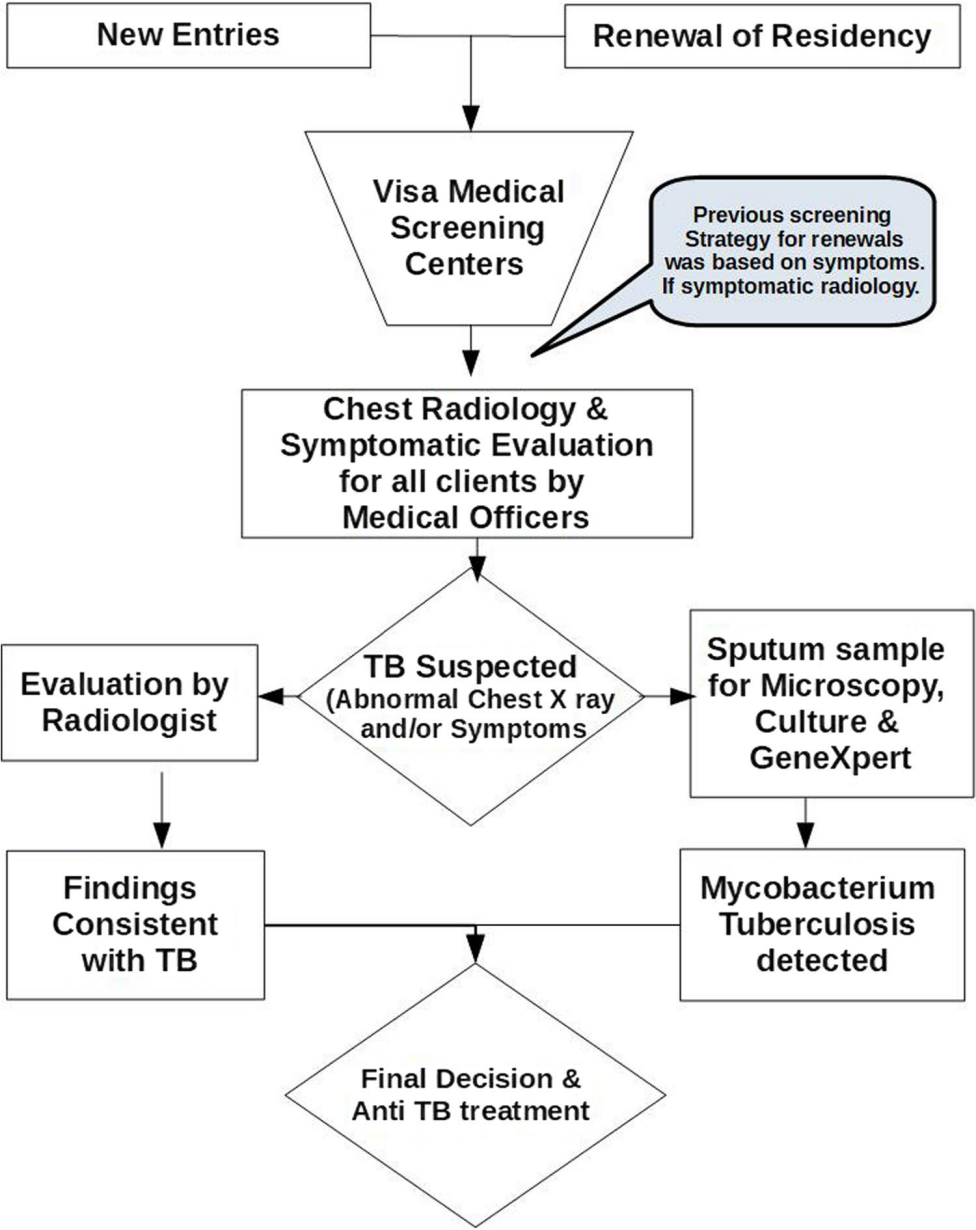
Screening algorithm. Attached as jpeg file

In 2018, the policy was changed to a specific algorithm (Fig. 1) in which all applicants including renewal of residency were required to undertake chest X-ray screening also in addition to symptom screening. Thus whether symptomatic or not all with suspected radiological findings were also subjected to sputum-smear microscopy, GeneXpert *Mycobacterium* tuberculosis (MTB)test (Cepheid, Sunnyvale, California, USA) and culture with both BACTEC MGIT 960 System(Becton–Dickinson, Sparks, MD, USA) and Lowenstein-Jensen medium. Radiological evaluations are conducted as follows; first, any chest X-ray abnormality are identified and flagged, and the chest X-Ray is referred to a radiologist; second, the radiologist consults and may order further evaluations such as a computerized tomography scan.

The target population for this study represents expatriates seeking medical fitness evaluations in Muscat Governorate either for their first residency application or for renewal of their residency done every 2 years. Oman is divided administratively into 11 governorates, and the capital is Muscat Governorate which also has a large population, representing approximately one-third of Oman’s population. Muscat Governorate having a population of approximately 1.3 million of which 58% are expatriates, accounts for 60% of medical screening of expatriates for visa related processes in Oman [6].

### Study Subjects and Data Collection

The study included subjects who had radiological suspicion of TB upon screening between November 2018 and January 2020 after which the screening process was suspended due to the COVID-19 pandemic. Data collection included information on demographics, relevant clinical history and medical examination findings, radiological findings, sputum microscopy, molecular test (GeneXpert) and culture results. This information along with number of subjects attending the screening process and their visa status was obtained from the electronic records system hosted by the Ministry of Health. We received ethical approval from our institutional research committee constituted under Ministry of Health. No personal identifiers of subjects participating in the study was used and data extraction was done from records maintained by the clinics and computerized records.

### Definitions Used for the Study

A new entrant is an expatriate who is applying for a first time residency visa and renewal those applying for renewal of existing residence visa. Applicants for residency visas are grouped under two categories based on occupation and related risk of infection. Category-1 includes occupations such as healthcare workers, beauty salon workers, domestic workers, housemaids and food handlers. Category-2 includes all other professions and family dependents (close family members of the expatriate who seeks employment). For presentation of findings, nationalities were grouped by six WHO regional offices, individual nationalities and based on TB burden as per 2020 Global TB report. Countries with TB rate of more than 100 per 100,000 were classified as high burden. A confirmed case is one which has been proven either by TB culture or by molecular testing (GeneXpert) [11]. For the study, symptoms were defined as those symptoms suggestive of TB disease such as cough lasting longer than 2 weeks, hemoptysis, weight loss, fever or night sweats [11].

### Statistical Analysis

Data were entered in an Excel (Microsoft Corporation, Redmond, Washington DC, USA) spreadsheet and the analysis was performed in Excel, OpenEpi (Open Source Epidemiologic Statistics for Public Health, www.OpenEpi.com) and SPSS Version 21 (IBM, Armonk, New York, USA). A descriptive analysis of demographic and clinical data was performed using appropriate statistical measures with 95% confidence intervals. Statistical significance was set at *P* = 0.05. TB prevalence estimates and confidence intervals were calculated for the study population and select subgroups. TB prevalence in our study population was compared to WHO-estimated prevalence in countries of origin in 2019, rates were calculated for the 15 to 65 age group, as expatriates screened belong to this group. Utilizing published data on TB prevalence in Oman [16], comparison of TB detection rates among expatriates by passive case detection and through active screening from 2013 was attempted to evaluate the change due to the changed strategy in active screening process in 2018. Variables that had previously been reported to be associated with TB in expatriate populations were analyzed for association with bacteriologically confirmed TB in the study population using chi square tests. Crude and adjusted odds ratios with 95% confidence interval were estimated for statistically significant and operationally relevant risk factors.

## Results

A total of 501,290 visa applicants were screened in Muscat Governorate during the study period. Among them 186,046 (37.1%) were new entries and 315,244 (62.9%) were renewing residency. Screening of these applicants with addition of Chest X-ray into the screening process resulted in 436 (0.09%) having radiological findings suggestive of active TB disease which translated into 1,150 chest X-rays to detect one abnormal (Table 1). Yield of chest X-ray screening nationality wise and by WHO country groupings are summarized in Table 1. Of 436 cases, 53 (12.2%; 95% CI 9.2–15.6) were confirmed by GeneXpert and/or culture positive for MTB. This gives an overall prevalence of 10.6 (95% CI 8–13.9) per 100,000 applicants (number needed to be screened 9458).Age-standardized TB rates corresponded to the rates of countries of origin, and higher rates among new entrants were noted from African countries compared to other countries (Table 2).

**Table 1:** Overall screening outcomes (applicants screened for chest X-ray abnormality and yield with number needed to be screened [NNS] to get one case with chest X-ray suggestive of TB)

Countries grouped by WHO Regional Organization groupings/countries with suspect cases	Number of applicants screened			Chest X-ray abnormality		Confirmed by bacteriology
	New	Renewal	Total	#	NNS	
Total	186,046	315,244	501,290	436	1150	53
African Region Organization (AFRO)	12,336	10,031	22,367	38	589	8
Eastern Mediterranean Region Organization (EMRO)	29,704	48,226	77,930	64	1218	8
European Region Organization EURO	3185	2922	6107	5	1221	0
Pan American Health Organization (PAHO)	1054	1004	2058	2	1029	0
South East Asia Region Organization (SEARO)	127,835	237,200	365,035	253	1443	29
Western Pacific Region Organization (WPRO)	11,932	15,861	27,793	74	376	8
By countries						
India	94,167	154,745	248,912	166	1500	22
Philippines	11,003	15,285	26,288	73	360	8
Bangladesh	25,007	72,801	97,808	69	1418	7
Pakistan	16,449	34,408	50,857	49	1038	6
Africa*	12,336	10,031	22,367	38	589	8
Sri Lanka	4957	4577	14,491	10	1449	0
Indonesia	419	1623	2461	5	492	0
Morocco	756	941	2453	5	491	1
Turkey	761	537	2059	5	412	0
Egypt	4816	5820	15,452	4	3863	1
Nepal	3043	3160	9246	3	3082	0
Iran	3348	1024	7720	2	3860	0
Others	8984	10,292	19,276	7	2754	0

*All African countries except Egypt and Sudan. No African countries other than Tanzania had GAMCA approved centers in 2018–19

**Table 2:** Comparison of the prevalence of active TB in expatriates screened to the WHO-estimated prevalence of active TB in 2019, for the ten nationalities having highest incidence in the study

Country	Country estimates with 95% confidence limits (per 100,000 population, 15–65 age group)^a^			TB rate per 100,000 with 95% confidence limits from study (15–65 age group)^b^				
(# of active cases detected)				Total (new + renew)			New	Renewals
	Rate	Lower	Upper	Rate	Lower	Upper	Rate	Rate
Bangladesh (7)	292.81	291.8	293.8	7.17	3.14	14.4	8.00	6.88
Burundi (1)	176.65	173.4	180.0	**72.52**	1.98	403	90.17	0.00
Egypt (1)	17.69	17.4	18.0	9.47	0.30	46.47	20.81	0.00
India (22)	247.7	247.5	248.1	8.89	5.18	12.6	4.25	11.73
Nigeria (1)	322.96	321.9	324.0	**36.83**	0.86	205	49.65	0.00
Pakistan (6)	367.63	366.6	368.7	11.87	4.08	25.99	12.18	11.73
Philippines (8)	750.49	748.5	752.5	30.49	12.86	59.86	27.29	32.80
Sierra Leone (1)	441.96	435.9	448.1	**120.34**	3.29	668.6	153.14	0.00
Tanzania (3)	313.42	146.8	429.1	**45.21**	9.54	131.8	59.26	30.67
Uganda (1)	302.72	300.5	304.9	**29.84**	0.47	166	79.68	0.00

African countries without pre-entry screening given in bold

^a^Data source: https://www.who.int/data/gho/data/themes/topics/topic-details/GHO/cases-and-deaths

^b^Number of cases among a nationality/total applicants screened with chest X-ray of that nationality

The proportion of active TB detected among those with abnormality in chest X-ray screening did not show any statistical difference between residents for renewal (14.04%, CI 10.2–19) and new entrants (9.95%, CI 6.5–14.9). Among those with an abnormal chest X-ray, 16 were smear positive, 45 were GeneXpert positive and 44 were culture positive. Comparing positivity between GeneXpert and TB culture 36 were positive for both, 9 were culture negative with GeneXpert positive, 8 were culture positive with GeneXpert negative. None was diagnosed with TB on clinical grounds alone.

Twenty-three among 436 with radiological findings had symptoms, of which 11 had active TB. Of the active cases detected, 33/53 (62.3%) were from applicants attending medical examination for renewal of visa and they had stayed in Oman at least 2 years. This gives a prevalence of 10.5 per 100,000 expatriates in the renewal group (95% CI 6.9–14.04 per 100,000 expatriates screened). Among the residents examined for renewal (follow-up), the duration of stay in Oman ranged from 2 to more than 43 years (mean, median and mode was 11.8, 9 and 2, respectively). Among the applicants for renewal, mean duration of stay in Oman for those diagnosed with TB (33/235) was 8.5 years compared to 10.7 for those not diagnosed (202/235), but was not statistically significant.

**Chart 1:**
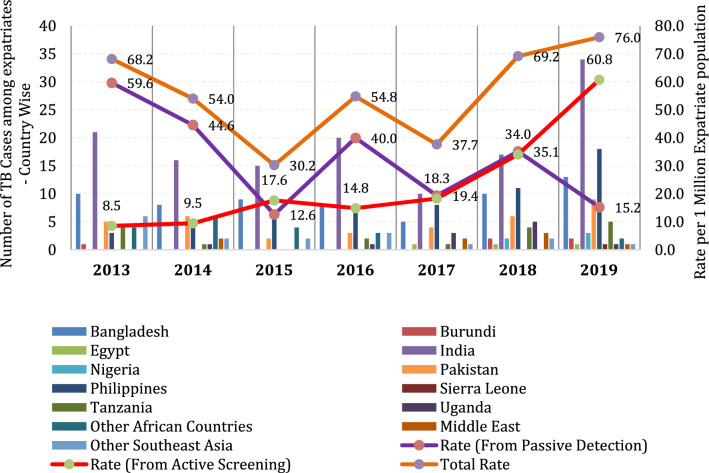
Trend in active TB case detection over past years and change with new screening policy in 2018

Radiological findings were segregated into two classes, those findings suggestive of active pulmonary TB and the other with fibrotic or lesions suggestive of previous lung disease (Table 3). Baseline demographic characteristics of applicants who were detected to have radiological features suggestive of TB are summarized in Table 4. The majority of those diagnosed with active TB were expatriates 26–45 years of age (66%), males (69%) and people coming from high TB burden countries (96%) (Table 4). Multivariate analysis (Table 5) with duration of stay in Oman, job categories, presence of symptoms and past TB disease history showed job category and presence of symptoms as independent predictors in this study population. High TB burden countries, absence of pre-arrival screening, job category, and presence of symptoms were likely factors that increased probability of TB in the studied population. Comparison of active and passive TB detection rates among expatriate population in the country since 2013 are given in Chart 1. The new strategy increased proportion of cases detected by active screening to above 80% compared to 10–50% during the period prior to it.

**Table 3:** Descriptive summary of radiological findings

Fibrotic lesion and old healed TB findings (144 applicants)	All	TB	Finding suggestive of active lung infection (292 applicants)	All	TB
Fibrous lesions	68	5	Reticulonodular and nodules	119	10
Fibro atelectasis	17	0	Opacities including calcified	116	19
Pleural thickening	22	2	Consolidation	21	8
Granulomas	12	0	Cavitation	19	4
Calcifications	12	2	Pleural effusion	9	1
Bronchiectasis changes	5	0	Infiltrates	12	2
Volume loss	5	0	Lymph nodes and hilar mass	5	0
Costophrenic blunting	2	0	Others	7	0
Others	9	0			
Diaphragmatic abnormalities	3	0

**Table 4:** Characteristics of the study cohort and incidence of TB

Characteristics (*n*)		TB detected (*n*, %)		*P* value
		Yes (53/436)	No (383/436)	
Age groups	13–25 (37)	7 (18.9)	30 (81.1)	0.2053 (degree of freedom = 2)
	26–45 (272)	35 (12.9)	237 (87.1)	
	46–85 (127)	11 (8.7)	116 (91.3)	
Chest X-ray	Fibrotic lesion and other old TB lesion findings (144)	9 (6.3)	135 (93.7)	0.006
	Finding suggestive of active lung infection (292)	44 (15.1)	248 (84.9)	
Gender	Female (169)	16 (9.5)	153 (90.5)	0.17
	Male (267)	37 (13.9)	230 (86.1)	
Country groups by pre-arrival screening	No pre-arrival screening (29)	4 (13.8)	25 (86.2)	0.96
	GAMCA screening present (407)	49 (12)	358 (88)	
Country group by TB burden	Low (35)	2 (5.7)	33 (94.3)	0.35
	High (401)	51 (10.2)	350 (89.8)	
Job-category	Category-2 (192)	31 (16.1)	161 (83.9)	0.024
	Category-1 (244)	22 (9.0)	222 (91)	
Resident status	New (201)	20 (10)	181 (90)	0.19
	Renewal (235)	33 (14.0)	202 (86)	
Past history of TB treatment	Yes (63)	3 (4.8)	60 (95.2)	0.06
	No (373)	50 (13.4)	323 (86.6)	
Duration in Oman	New entrant (201)	20 (10)	181 (90)	0.41 (degree of freedom = 3)
	2≤ 4 years (46)	6 (13.0)	40 (87)	
	4≤ 6 years (24)	5 (20.8)	19 (79.2)	
	≥ 6 years (165)^$^	22 (13.3)	143 (86.7)	
Symptoms	No (413)	42 (10.2)	371 (89.8)	< 0.005
	Yes (23)	11 (47.8)	12 (52.2)	
BCG (*n* = 409)	Present (259)	30 (11.6)	229 (88.4)	0.28
	Absent (150)	23 (15.3)	127 (84.7)	
BMI (*n* = 408)	Normal (227)	33 (14.5)	194 (85.5)	0.01* (degree of freedom = 3) *one cell value < 1
	Overweight (105)	7 (6.7)	98 (92.3)	
	Obese (38)	0 (0)	38 (100)	
	Underweight (38)	7 (18.4)	31 (81.6)

**Table 5:** Risk factors associated with TB diagnosis in the study population from multivariate analysis

Associated risk factors (#)		TB detected (53/436)	Odds ratio with 95% confidence interval	
			Crude	Adjusted
Job-category	Category-2 (192)	31 (16.1)	1	1
	Category-1 (244)	22 (9.0)	0.52 (0.29–0.92)	0.39 (0.16–0.96)
Past history of TB treatment	Yes (63)	3 (4.8)	1	1
	No (373)	50 (13.4)	0.32 (0.08–0.97)	0.47 (0.10–2.1)
Symptoms	No (413)	42 (10.2)	1	1
	Yes (23)	11 (47.8)	8.1 (3.37–19.49)	13.5 (3.96- 45.5)
Duration of stay (for each additional year of stay)			0.97 (0.92–1.02)	0.95 (0.9–1.01)

## Discussion

Towards achieving the ambitious target of TB elimination, one key strategy adopted in Oman was to strengthen the visa medical screening process within the country through a revised active TB-screening algorithm triggered by radiological suspicion and not the previous symptom-based trigger alone. This study describes the findings after a year of its implementation.

A higher proportion of cases (see Tables 1 and 2) were observed in new entrants from African countries such as Burundi, Uganda, Sierra Leone and Nigeria who had no pre-arrival screening facilities in their country. Estimated prevalence rates by nationality in the study group reflected to some extent the disease burden of their country of origin especially among new entrants (see Table 2). Similar findings were reported from other GCC countries as well as from the United Kingdom and Australia [8, 17–21]. Screening strategies adopted by low-burden countries have substantial heterogeneity in screening criteria and the screening algorithms [22–24]. Oman’s strategy of pre-arrival screening, screening at entry, and follow-up screening provides a robust strategy with potential for strengthening [17, 19–21]. The differences in rates especially between new entries and renewals as well as among nationalities (see Tables 1 and 2) could be indicative of socioeconomic differences between the expatriate population in Oman, prevalence rates in countries of origin and possibly other confounders [24, 25]. An important confounder is the heterogeneity in pre-arrival screening status with some applicants having no pre-arrival screening and some older applicants re-entering as new entrants after a gap of a few years.

The new algorithm made active screening for detecting TB among visa applicants more efficient compared to the symptom-based process used before which is reflected in the increased proportion contributed by active case detection in TB incidence rate since 2018 (Chart 1). The increasing trend in cases detected over the past 7 years and the increase noted by the new active screening process over the previous passive process supports validity of pre- and post-migration screening reports from other low-burden countries [17, 18, 22]. Given that only 11 out of 53 active cases detected had symptoms, it can be assumed that by utilizing the combination of chest X-ray with Xpert MTB tests in screening, an additional 42 cases were detected.

In a country like Oman, when opting for latent tuberculosis (LTBI) screening and treatment for expatriates, our study findings support the need for screening before arrival. Chest radiography is an important tool for TB triaging and screening, the major disadvantage is that it requires experienced staff. As a radiograph is highly sensitive but less specific to identify TB, rigorous efforts need to be made to follow-up on suspect findings with bacteriological confirmation (sputum-smear microscopy and culture or a molecular test) [11, 26]. From 2011 to 2015, the International Organization for Migration conducted health assessments for more than 1.5 million immigrants and refugees and reported that 5% had chest radiography findings consistent with TB, and among those 7% were diagnosed with TB [26]. We need to note that there is much heterogeneity in reasons for migration to various countries. It is important to highlight that in this screening strategy adopted by Oman, the positive predictive value will always tend to be on the lower side being a low prevalence country and because of the multilevel screening process (pre-entry, post-entry and renewal of visa) for entry of expatriates [17, 19, 21, 26].

TB in migrants comprises either reactivation of latent TB infection diagnosed some years after entry or cases missed by pre-entry or at-entry screening. It is often held that new cases of TB that occur some years after entry could be the result of the reactivation of LTBI [8, 13]. It is valid, therefore, to examine the TB rates among the sub group of applicants who were screened for renewal and can be considered as follow-up screening [25]. Our estimated TB incidence of 10.6 on follow-up (among renewals) is very much less than the post-migration follow-up incidence reported in literature [25, 27]. This could be indicative of the difference in the setting of the screening, quality of the process as well as due to exclusion of the symptomatic cases diagnosed in healthcare settings. When we examine the active TB cases detected in our study, the maximum number of cases was detected within 4 years of entry, indicating that screening aimed only at identifying active TB cases at entry may miss an unknown proportion of cases who are most at risk to develop TB. It is important to note that screening for active TB at entry detects only a small number of TB cases and will miss cases that can reactivate after entry if screening for LTBI is not included [12, 13, 15, 28, 29].

From a forward-looking perspective, three observations emerge from our findings:

First, it is important to continue screening after arrival even in subjects where the initial exit screening from country of origin gave no suspicion of TB.

Second, there is a potential to further refine the algorithm to increase the efficiency of the follow-up screening incorporating other risk factors such as country of origin, as well as that of radiological screening strategies by use of artificial intelligence (AI)-enabled tools.

Third, that TB diagnosis among expatriates many years after entry may also be due to re-exposure by frequent revisits to their countries of origin, a fact that has a bearing when implementing a LTBI screening policy [28–30].

Our study has several limitations. It was conducted from an operational perspective and there is unmeasured yet possibly small, risk of misclassification of the X-rays during radiological evaluation, although the criteria were that any suspected radiological abnormality indicated screening for TB. In comparing rates with countries of origin, an element of sampling bias is expected as each nationality represents different socioeconomic backgrounds and because the individual expatriates cannot be considered to strictly represent the population of their country of origin. As mentioned before the heterogeneity in pre-arrival screening status, re-entry after a gap of a few years, visiting relatives in high TB burden countries are characteristics that makes the immigrant screening setting in Oman as well as GCC countries unique. This and other possible confounders make our study findings relating to risk factors for TB disease less generalizable to other settings. Mantoux or interferon-gamma release assays (IGRA) testing was not uniformly done for all the study subjects due to operational and policy issues. In this context, it is important that high Mantoux or IGRA positivity is expected in the study group, as reported by Yaquobi et al. In this study to estimate proportion of migrants to the Sultanate of Oman with LTBI, the overall IGRA-positive rate was 22.4% with 30.9% and 21.2% among African and Asian migrants, respectively, and would not add value to detection of bacteriology proven TB disease [30].

It is prudent to consider aspects not addressed in this study such as the costs involved, efficiency and predictive value of the screening process in context. Study findings will help in choosing an accurate screening algorithm critical for a country like Oman where nearly 40% of the population are expatriates with significant mobility. The detection of active cases in follow-up screening enhances the urgency for implementation of a policy for LTBI screening and management among expatriates and there is scope to customize it for specified high-risk populations. In addition, it is important to recognize the higher probability of re-exposure risks in expatriates while visiting their home countries and risk of overcrowded dwelling units in a segment of expatriates with low income. In this regard, studies relating to cost-effectiveness of identifying and treating LTBI among migrants from high-endemic countries to low endemic countries indicate that strategies need to be carefully studied for cost-effectiveness, suitability for the country setting and demographic characteristics of the immigrants [28–31]. An additional important recommendation would be to establish a post-migration follow-up algorithm for people identified during visa screening as having an increased risk of developing TB later.

## Conclusion

Our study findings of increased detection rate supports the utilization of chest X-ray as a preferred tool for active case finding in migrant screening compared to previous symptom-based algorithm. This preference is enhanced by other factors such as availability of digital radiography with its advantages, better radiation safety and the increasing availability of rapid molecular tests with higher sensitivity and specificity [11, 26]. Exit screening from country of origin results in lower pre-test prevalence than the group without exit screening. It is also evident that higher prevalence in countries of origin is a risk factor for diagnosis of TB at entry or upon follow-up. It is important to have follow-up screening in subjects who show no sign of TB at the initial “at entry” screening.

## Data Availability

The data collected for the purpose of this study are owned by Ministry of Health and will be available for purposes with specified intent.

## Abbreviations

TB: Tuberculosis
CI: Confidence interval
WHO: World health organization
GCC: Gulf cooperation council
OECD: Organization for economic co-operation and development
GAMCA: Gulf approved medical centers association
HIV: Human immunodeficiency virus
MTB: Mycobacterium tuberculosis
USA: United States of America
SPSS: Statistical package for the social sciences
IBM: International Business Machines Corporation
LTBI: Latent tuberculosis infection
IGRA: Interferon gamma release assay
